# Treatment Planning Methods in High Dose Rate Interstitial Brachytherapy of Carcinoma Cervix: A Dosimetric and Radiobiological Analysis

**DOI:** 10.1155/2014/125020

**Published:** 2014-01-23

**Authors:** Surega Anbumani, Pichandi Anchineyan, ArunaiNambiraj Narayanasamy, Siddanna R. Palled, Sajitha Sathisan, Punitha Jayaraman, Muthu Selvi, Ramesh S. Bilimagga

**Affiliations:** ^1^Department of Radiation Oncology, HCG Bangalore Institute of Oncology, RRMR Extension KH Road, Bangalore 560027, India; ^2^Photonics, Nuclear and Medical Physics Division, VIT University, Vellore 632014, India; ^3^Department of Radiation Oncology, Kidwai Memorial Institute of Oncology, Hosur Road, Bangalore 560029, India

## Abstract

Treatment planning is a trial and error process that determines optimal dwell times, dose distribution, and loading pattern for high dose rate brachytherapy. Planning systems offer a number of dose calculation methods to either normalize or optimize the radiation dose. Each method has its own characteristics for achieving therapeutic dose to mitigate cancer growth without harming contiguous normal tissues. Our aim is to propose the best suited method for planning interstitial brachytherapy. 40 cervical cancer patients were randomly selected and 5 planning methods were iterated. Graphical optimization was compared with implant geometry and dose point normalization/optimization techniques using dosimetrical and radiobiological plan quality indices retrospectively. Mean tumor control probability was similar in all the methods with no statistical significance. Mean normal tissue complication probability for bladder and rectum is 0.3252 and 0.3126 (*P* = 0.0001), respectively, in graphical optimized plans compared to other methods. There was no significant correlation found between Conformity Index and tumor control probability when the plans were ranked according to Pearson product moment method (*r* = −0.120). Graphical optimization can result in maximum sparing of normal tissues.

## 1. Introduction 

Cervical cancer is a major cancer burden which constitutes number one among Indian women with relative survival rate of 48.7% [1]. EBRT followed with brachytherapy (BT) is the standard of care and an integral part of local control of the disease. Cervix carcinoma has been treated with HDR BT for more than 30 years. It has advantages in terms of local recurrence, mortality, and late complications for the clinical stages I, II and III, similar to those of Low Dose Rate therapy [2]. The present standard of care using concomitant chemotherapy and radiotherapy has resulted in 80–90% local control rates for early stages [3–5]. But a decline of about 67–75% is noted for advanced stages because of the local failure due to inadequate dose coverage [6–8].

Interstitial brachytherapy (ISBT) is best suited for the patients with anatomy not allowing for standard intracavitary application and wherein the disease could not be encompassed in the standard ICBT application. It can improve the dose coverage with various normalization and optimization techniques. Improved planning strategies and dose optimization can reduce normal tissue complications rate without compromising local control of the disease [9]. Clinical investigations and dosimetric comparisons in the evaluation of interstitial implants are scarce and heterogeneous. Available data are from limited number of patients. Thus, the potential advantage of ISBT in gynecological malignancies could not be clearly demonstrated [10]. Interstitial implants, two dimensionally planned with semiorthogonal films, were radiobiologically evaluated using a figure of merit (FOM) and biologically effective dose by Supe et al. [11]. Different optimization techniques used in computed tomography based planning were dosimetrically compared in 10 patients by Shwetha et al., in which dose normalization methods were not considered [12]. The authors from Tata Memorial Hospital, India, have dosimetrically compared the anatomy based inverse planning with the graphical optimization for five prostate cases [13]. The studies conducted were on lesser number of patients and evaluated with mean and standard deviation values which could not establish a statistically significant result. None of these studies comprehensively evaluated dosimetrical and radiobiological indices.

HDR brachytherapy has the advantage of calculating dose distributions by altering radioactive source dwell times and dwell positions using sophisticated planning methods. Dose point normalization (DP_N) is a procedure where dose can be normalized to prescription dose along the target outline using dose points defined. The prescribed dose is optimized in the earlier defined dose points oriented to the target's surface in the dose point optimization method. Reference isodose line can be normalized to a 5 mm box surface of the needle implant in geometrical normalization. In Geometrical Optimization procedure, dwell weight of each catheter position is primarily influenced by active dwell positions on other catheters, therefore filling in the spaces to cover desired volume. Dose optimization algorithms in general are said to be effective in optimizing the dose distributions by reducing the high dose regions inside the target volume. But the clinical advantage of each optimization technique should be evaluated.

Geometrical optimization was the previously followed ISBT planning technique in our institution. Radiation dose can be normalized using dose point and geometrical normalization techniques. The graphical optimization was compared with DP_N, DP_O, G_N, and G_O. In this study we have evaluated the ISBT planning methods for 40 patient cases with seven dosimetric parameters [14]. In addition to dosimetric analysis, the cervix implants were assessed with TCP and bladder and rectum NTCP estimates formulated by Kehwar et al. based on biologically effective equivalent uniform dose concept. Thus, the optimal ISBT planning method to improve local control of cervical cancer can be suggested.

## 2. Methods 

40 cervical cancer patients (stage IIB/stage IIIB) treated with High Dose Rate Microselectron unit (V2) were selected for this retrospective study. Interstitial needles were inserted using vaginal obturator (VO) in the template (either Nucletron MUPIT or Syed Neblett) under general anesthesia through transperineal route. First a guide needle is inserted into the anterior lip of cervix and the other 5 needles were inserted into the groove of the VO. The total number of needles used ranges from 20 to 26 depending upon disease extension. During the implant procedure, utmost care was taken in order not to perforate bladder and rectum with the needles. Patients with needles in situ were CT scanned (slice thickness 5 mm) and the images were converted into DICOM RT files. The CT images were acquired into Plato Sun Rise Treatment Planning System Version v 3.5.1. In the virtual simulation module of Plato Version VSS v1.6 (Nucletron, Elekta AB, The Netherlands), target volume and critical structures such as bladder and rectum were delineated by the physicians (clinical oncologists). Catheters were reconstructed in the brachytherapy planning module BPS v 14.3. In our institution, dose point based optimization followed by graphical optimization is the planning method in practice. Dose points for normalizing the prescribed dose (6 Gy per fraction) were created at an interspace distance of 5 mm on the target volume. Only those dwell positions of the source with step size 2.5 mm which encompass the target volume alone were activated manually. Then the dose points were normalized to prescription dose and then optimized. Further optimization by manual dragging of isodose lines was done with the graphical optimization tool. It is used to customize the shape of an isodose distribution along the target volume. It allows the manipulation of isodose curves and modifies the individual dwell weights or dwell times by dragging the isodose lines visualized in the axial CT sections. This is a trial and error process in which the dwell weight/dwell time are adjusted to achieve an optimal target coverage with less bladder (D2 = 5 Gy) and rectum doses (D2 = 4 Gy). Hence it requires a very high expertise of the planner. Other available planning methods in Plato BPS which need to be validated and compared [15].

CT scans of 40 patients previously treated with Gr_O were used to construct hypothetical treatment plans using DP_N, DP_O, G_N, and G_O. Hence, all the 40 patients planned with graphical optimization technique (Gr_O) were kept as their own internal control in this study. Dose volume histograms (CTV, Bladder, and Rectum) for each patient with five plans were generated that resulted in a total of 200 (40 × 5) plans and a sum of 600 DVHs (200 × 3). The dosimetric parameters such as TVDref, TV1.5Dref, TV2Dref, D2cc (Bl), and D2cc (R) were extracted from the DVHs. From these parameters, the dosimetric quality predictors such as Coverage Index (CI), External Volume Index (EI), Dose Homogeneity Index (DHI), Overdose Volume Index (ODI), and Dose Nonuniformity Index (DNR) defined by Meertens et al. were computed (Table 1):

(1) $$\begin{array}{c} \text{CI}=\frac{\text{TVDref}}{\text{TV}}\\ \text{EI}=\frac{\text{NTVDref}}{\text{TV}}\\ \text{DHI}=\frac{\left[\text{TVDref}-\text{TV}1.5\text{Dref}\right]}{\text{TVDref}}\\ \text{ODI}=\frac{\text{TV}2.0\text{Dref}}{\text{TVDref}}\\ \text{DNR}=\frac{\text{TV}1.5\text{Dref}}{\text{TVDref}}. \end{array}$$

CI is the fraction of the target volume that receives a dose equal to or greater than reference dose. EI is defined as the ratio of the volume of normal tissue that receives a dose equal to or greater than reference dose to target volume whereas DHI is the ratio of target volume receiving a dose in the range of 1.0 to 1.5 times of reference dose [16, 17]. ODI is the ratio of the target volume which receives a dose equal to or more than 2.0 times of the reference dose. DNR is calculated from the ratio of the target volume which receives a dose equal to or greater than 1.5 times of reference dose [18]. For an ideal implant, CI is 1, EI is 0, DHI is 1, ODI is 0, and DNR is 0.

Radiobiological indices such as TCP and NTCP were calculated from DVH data based on Kehwar et al. using an indigenously developed Matlab program [18]. The software program was cross-validated with an independent Excel calculation. Parameters for bladder and rectum from Emami et al. data were used as the input to calculate NTCP values.

### 2.1. Statistical Analysis

The *P* values for the dosimetric and radiobiological indices were calculated using paired Student's *t* test using statistics computation environment STATISTICA 5.0 (StatSoft Inc., USA). Confidence intervals were evaluated using the *P* values. Pearson product moment correlation method was used to correlate CI with TCP estimates. The correlation coefficient *r*(*x*, *y*) was computed. The 2D scatter plot demonstrating the correlation was generated.

### 2.2. Results

G_O resulted in a higher CI with a mean value of 0.8879 compared to the graphical optimized plan (*P* = 0.0001). EI was less in Gr_O plans (mean = 0.0009). Homogeneity of dose distribution is higher for Gr_O and DP_N plans which is obvious from the mean DHI values 0.6629 and 0.6282, respectively, whereas other methods resulted in a lesser homogeneous dose inside the target (Table 1). ODI and DNR were less for Gr_O and DP_N methods. The dose to 2cc volumes of bladder and rectum were minimized with Gr_O method (B2cc = 4.25 Gy; R2cc = 4.16 Gy). Dose point normalization resulted in a lesser bladder dose (mean 4.61 Gy) which is comparable to Gr_O plans. TCP computed was found to be similar for all the five planning techniques with no statistically significant results. NTCP for bladder (mean: 0.3252%) and rectum was less (mean: 0.3126%) for Gr_O plans whereas G_O plan resulted in high NTCP values (Table 1).

**Figure 1:**
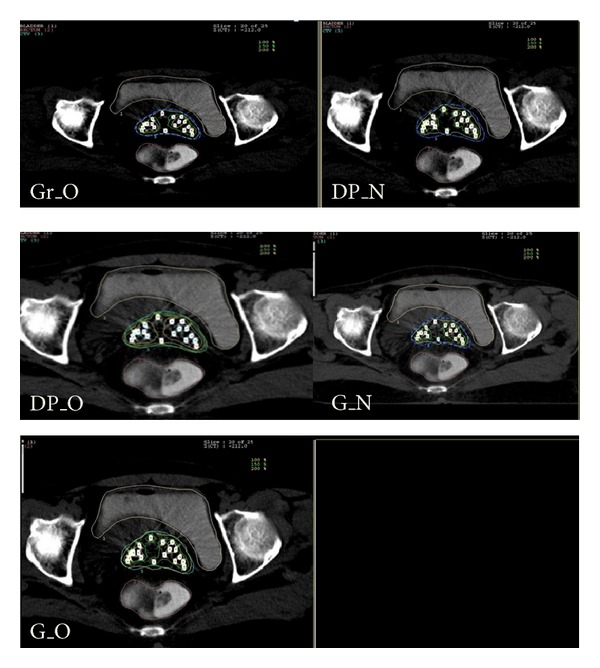
Comparative axial slice view of the ISBT plans.

**Figure 2:**
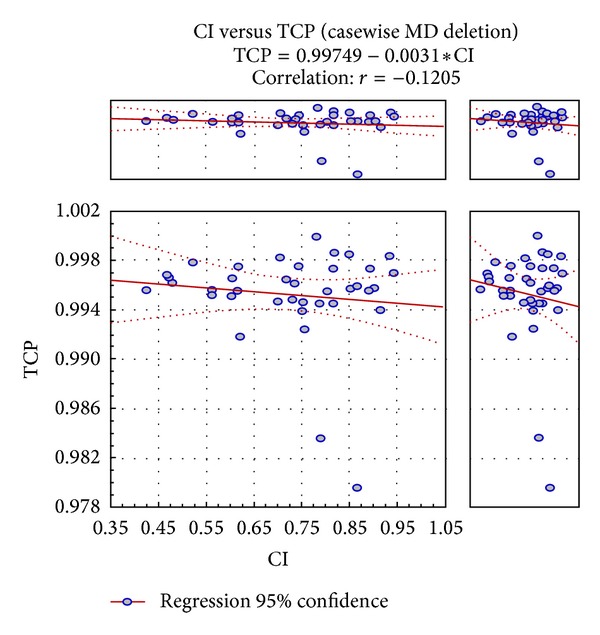
Graph correlating CI and TCP.

Dose distributions resulted from each method are analysed using the axial CT sections (Figure 1). The high dose volumes (>100% of the prescribed dose) were found to be less for Gr_O and DP_N plans compared to other plans. Gr_O plan had smaller volumes of higher isodose regions (150% and 200%) only around the needles.  Figure 2 shows the scatter plot diagram of the Pearson product moment correlation between CI and TCP. The regression coefficient (*r*) computed was −0.120 which is not statistically significant (confidence interval: 95%).

### 2.3. Discussion

HDR BT often results in heterogeneous dose distribution owing to the steep dose falloff in the vicinity of the radioactive source. But a uniform dose is required to achieve the optimal tumor cell killing avoiding the necrosis. Normalization and optimization algorithms are being used for dose calculation in CT based planning of cervix implants. These methods are said to improve the dose homogeneity within the tumor. Geometrical optimization of dose distributions depends only on the implant volume. A template guided interstitial implant procedure done without proper image guidance could encompass the normal structures. Hence it increases the risk of normal tissue complications. In dose point optimization, reference isodose line is optimized along predefined dose points (interspace distance: 3 mm) on the target's surface. Bladder and rectal volumes are proximal to the delineated cervix. Hence dose optimized to surface of target volume encompasses the normal structures. graphical optimization helps in dragging the isodose lines that resulted from dose point optimization reducing critical organ doses. Gr_O is a powerful optimization tool which allows the planner to drag the isodose line to a desired level. Thus dwell weights/times are adjusted accordingly to meet the dose constraints. Appropriate and efficient use of the tool is still a learning curve for the planners. Efficiency of optimization and normalization techniques needs to be quantified to choose the optimal one for achieving better clinical outcomes.

The current research report that is the first one to evaluate interstitial brachytherapy plans with dosimetrical and radiobiological indices (TCP and NTCP) was performed for a larger set of DVH data (*n* = 600). Tumor control and normal tissue complication probability estimation using linear quadratic formula devised by Kehwar et al. for interstitial implants were used. Voxel element calculations were used to account for high dose gradients in the HDR implants. A better dose homogeneity can be achieved with the graphically optimized and volume normalized plans compared to others. But the normalization around the implant geometry (geometrical normalization) had resulted in more inhomogeneous dose distribution (mean DHI = 0.4494). Target dose homogeneity was maintained in Gr_O plans. Overdose Volume Index (ODI) and Dose Non uniformity Ratio (DNR) showed no statistically signiicant diference between Gr_O and DP_N methods. The External Volume Index quantifying the normal tissue dose outside target volume is very less for graphically optimized plans with an extreme statistical significance with other methods. Bladder and rectal volumes usually remain very close to the target volume in cervical cancer. Conformity Index was compromised with the manual pulling of isodose lines from the critical organs which typically engross the target volume. The dosimetric analysis of target coverage with dose volume parameters may imply inadequate target coverage with graphical optimization. But it may not be influenced by the tumor control probability since there was no correlation with Conformity Index. TCP estimates were comparable to those of normalization and optimization methods. Thus the use of graphical optimization in increasing tumor control and reducing bladder and rectal toxicities can be established.

CI and TCP cannot be correlated because of the statistically insignificant regression coefficient (Pearson product moment correlation). The *P* values computed for the TCP estimates had no statistical difference. When the dose point (surface) optimized dose distributions are graphically or manually optimized, the dose to the 2cc volume of bladder and rectum can be reduced well compared to other automated dose calculation methods with extremely good statistical significance (95% confidence interval). Fairly good normal tissue complication probability estimates were also obtained for Gr_O plans. The use of graphical optimization in planning the interstitial cervix cases can spare the bladder and rectal doses to a greater extent without compromising the TCP estimates. Grade I and Grade II bladder and rectal complexities were observed in the clinical follow-ups (on treatment and posttreatment).

## 3. Conclusions 

graphical optimization is more beneficial in reducing radiotherapy side effects without compromising disease control. Hence it can be suggested as a method of choice for planning the interstitial cervix implant cases. The potential dosimetric results need to be corroborated with clinical outcomes.

## Figures and Tables

**Table 1:** Dosimetrical and radiobiological data for the ISBT planning methods.

Quality metric	Gr_O (mean)	DP_N (mean/*P*)	DP_O (mean/*P*)	G_N (mean/*P*)	G_O (mean/*P*)
CI	0.7320	0.7360 (0.87)	0.8561 (0.0001)	0.8356 (0.0004)	0.8879 (0.0001)
EI	0.0009	0.0174 (0.0001)	0.0114 (0.0035)	0.0441 (0.0001)	0.0406 (0.0001)
DHI	0.6629	0.6282 (0.1647)	0.6070 (0.049)	0.4494 (0.0001)	0.4764 (0.0001)
ODI	0.1343	0.1284 (0.5555)	0.1599 (0.0077)	0.1835 (0.0002)	0.1514 (0.0632)
DNR	0.3370	0.3717 (0.1647)	0.3929 (0.0049)	0.5205 (0.0001)	0.5535 (0.0001)
Bl2cc	425.65	461.72 (0.0697)	473.35 (0.0078)	524.37 (0.0001)	533.07 (0.0001)
R2cc	416.12	520.62 (0.0001)	504.95 (0.0001)	595.32 (0.0001)	620.47 (0.0001)
TCP	0.9952	0.9951 (0.0343)	0.9958 (0.5353)	0.9964 (0.0641)	0.9959 (0.9274)
NTCP-B	0.3252	0.3607 (0.0001)	0.7752 (0.0001)	0.7115 (0.0001)	0.8895 (0.0001)
NTCP-R	0.3126	0.7216 (0.0001)	0.7032 (0.0001)	0.8569 (0.0001)	0.8112 (0.0001)

CI: Conformity Index; EI: External Volume Index; DHI: Dose Homogeneity Index; ODI: Overdose Volume Index; DNR: Dose nonuniformity Ratio; Bl2cc: dose to 2cc bladder volume; R2cc: dose to 2cc rectal volume; NTCP-B: normal tissue complication probability of bladder; NTCP-R: normal tissue complication probability of rectum; DP_N: volume normalization; DP_O: dose point optimization; G_N: geometrical normalization; and G_O: geometrical optimization.

## Abbreviations

CT: Computed tomography
HDR: High dose rate
ISBT: Interstitial brachytherapy
Gr_O: Graphical optimization
V_N: Volume normalization
V_O: Volume optimization
G_N: geometrical normalization
G_O: Geometrical optimization
CI: Conformity Index
EI: External Volume Index
DHI: Relative Dose Homogeneity Index
ODI: Overdose Volume Index
DNR: Dose Nonuniformity Ratio
Bl_2cc _: Dose to 2cc volume of bladder
R_2cc_: Dose to 2cc volume of rectum
TCP: Tumor control probability
NTCP: Normal tissue complication probability.
